# Variation in Human Cytochrome P-450 Drug-Metabolism Genes: A Gateway to the Understanding of *Plasmodium vivax* Relapses

**DOI:** 10.1371/journal.pone.0160172

**Published:** 2016-07-28

**Authors:** Ana Carolina Rios Silvino, Gabriel Luiz Costa, Flávia Carolina Faustino de Araújo, David Benjamin Ascher, Douglas Eduardo Valente Pires, Cor Jesus Fernandes Fontes, Luzia Helena Carvalho, Cristiana Ferreira Alves de Brito, Tais Nobrega Sousa

**Affiliations:** 1 Molecular Biology and Malaria Immunology Research Group, Centro de Pesquisas René Rachou, Fundação Oswaldo Cruz (FIOCRUZ), Belo Horizonte, Minas Gerais, Brazil; 2 Biosystems Informatics Research Group, Centro de Pesquisas René Rachou, Fundação Oswaldo Cruz (FIOCRUZ), Belo Horizonte, Minas Gerais, Brazil; 3 Department of Biochemistry, University of Cambridge, Cambridge, United Kingdom; 4 Hospital Julio Muller, Universidade Federal de Mato Grosso, Cuiabá, Mato Grosso, Brazil; Agency for Science, Technology and Research - Singapore Immunology Network, SINGAPORE

## Abstract

Although *Plasmodium vivax* relapses are classically associated with hypnozoite activation, it has been proposed that a proportion of these cases are due to primaquine (PQ) treatment failure caused by polymorphisms in cytochrome P-450 2D6 (CYP2D6). Here, we present evidence that CYP2D6 polymorphisms are implicated in PQ failure, which was reinforced by findings in genetically similar parasites, and may explain a number of *vivax* relapses. Using a computational approach, these polymorphisms were predicted to affect the activity of CYP2D6 through changes in the structural stability that could lead to disruption of the PQ-enzyme interactions. Furthermore, because PQ is co-administered with chloroquine (CQ), we investigated whether CQ-impaired metabolism by cytochrome P-450 2C8 (CYP2C8) could also contribute to *vivax* recurrences. Our results show that CYP2C8-mutated patients frequently relapsed early (<42 days) and had a higher proportion of genetically similar parasites, suggesting the possibility of recrudescence due to CQ therapeutic failure. These results highlight the importance of pharmacogenetic studies as a tool to monitor the efficacy of antimalarial therapy.

## Introduction

Several factors have highlighted the importance of malaria caused by *Plasmodium vivax*, such as the spread of parasites that are resistant to available drugs [1]. In addition, the concept of vivax malaria as a benign disease has changed due to the description of severe cases and even deaths [2, 3]. Finally, dormant forms of the parasite that reside in the liver, i.e., hypnozoites, act as a reservoir for the disease and have hindered the control of malaria caused by *P*. *vivax*. Accordingly, it has been estimated that relapses cause between 50 and 80% of *P*. *vivax* infections in children living in areas with hyperendemic transmission [4–6].

There has been much speculation about *P*. *vivax* relapse and the factors responsible for its hypnozoite activation, including the saliva components of biting mosquitoes [7] and the systemic febrile illnesses associated with other parasitic or bacterial infections [8]. Additionally, the number of sporozoites inoculated by the anopheline mosquito may be an important determinant of both the timing and the number of relapses [9, 10]. In general, while parasites from tropical zones exhibit a short latent period before frequent episodes of relapse, parasites from temperate zones show a long latent period followed by few relapses [11]. Previous clinical studies showed that the majority of relapse episodes were caused by a parasite population distinct from the initial infection [12–14]. It has been proposed that in endemic areas, previous infections could also be a source of heterologous latent hypnozoites. Accordingly, a prospective infant cohort study in Thailand demonstrated that the first *P*. *vivax* relapses of life are usually genetically homologous [15]. It is also important to consider that malaria infection can be induced by the inoculation of more than one clone of sporozoites, and thus, genetically distinct hypnozoites can remain dormant until some are activated [12].

Beyond occurrence of the classical relapses caused by hypnozoite activation, recent findings suggest that some relapses could be due to the ineffectiveness of treatment with the drug used to kill the hypnozoites, primaquine (PQ) [16, 17]. PQ is the only FDA-approved drug that is currently indicated to treat relapsing strains of *P*. *vivax*, and its efficacy is thought to involve the formation of redox-active metabolites against the hypnozoites in the liver [18, 19]. The metabolism of PQ to its active metabolites has been shown to be dependent on cytochrome P-450 2D6 (CYP2D6) [18, 20, 21], which is an important member of the cytochrome P-450 superfamily responsible for the metabolism of approximately 25% of clinically used drugs [22]. Recently, it was demonstrated in both animal models and humans that decreased CYP2D6 activity has a significant effect on PQ metabolism and clearance [16, 17, 23]. The *CYP2D6* gene has a high allelic heterogeneity that results in great inter-individual variations in the level and activity of the enzyme [24, 25]. The number of functional gene copies of CYP2D6 is an important determinant of drug clearance for many substrates of this enzyme [26]. Subjects who have multiple gene copies (UM phenotype) will metabolize drugs more rapidly and, thus, have a potential risk of treatment failure because therapeutic plasma levels will not be achieved at the usual drug dosage [22]. However, for prodrugs such as PQ, it is possible that UM patients may derive a greater therapeutic benefit than other patients, but this has not been tested.

In this context, we sought to investigate the possible contribution of CYP2D6 variation to relapses of vivax malaria. Specifically, we evaluated the frequency of CYP2D6 polymorphisms associated with decreased enzyme function in two well-defined groups differing in their number of relapses (single- and multiple-relapses). Because these individuals were travelers who were returning home after visiting malaria transmission areas, it was an excellent opportunity to investigate the contribution of individual genetic variation to *P*. *vivax* relapses. Furthermore, using a computational approach, we predicted the effect of the identified CYP2D6 polymorphisms on enzyme stability and interaction with PQ.

In addition to relapse, *P*. *vivax* recurrence can be caused by reinfection or recrudescence as a consequence of blood-stage drug treatment failure. The drug that is commonly used to kill the blood-stage of *P*. *vivax* is chloroquine (CQ), which is metabolized mainly by cytochrome P-450 2C8 (CYP2C8) [27, 28]. For *P*. *falciparum*, mutations in CYP2C8 that confer a poor metabolizer (PM) phenotype may influence the parasite selection dynamics [29]. The CQ impaired metabolism may result in a longer CQ half-life and, thus, a longer parasite exposure to subtherapeutic levels of the drug. Here, to investigate the possibility that anti-schizonticidal therapy failure of CQ could also contribute to the observed *P*. *vivax* recurrences, we analyzed the polymorphisms in CYP2C8 that are predicted to determine a low CQ metabolizer phenotype.

## Materials and Methods

### Ethics statement

Ethical aspects of this study were approved by the Ethics Committee of Research involving Human subjects of Centro de Pesquisas René Rachou/Fiocruz (Protocol 377.205). All participants signed a written informed consent, including the next of kin, caretakers, or guardians on behalf of the minors/children enrolled in the study.

### Study area and subjects

A total of 46 patients who had relapsed *P*. *vivax* infections were selected (7–64 years old, median 33). Following treatment, the reappearance of parasitemia occurred once in 28 (60.9%) patients (defined as the single-relapse group) and two or three times in 18 (39.1%) (defined as the multiple-relapse group). The eligibility criteria included the following: (i) a relapsed non-complicated *P*. *vivax* malaria infection that had intervals between the initial episode and the relapse ranging from 29 days to 6 months; (ii) patients who were not re-exposed to malaria transmission during the interval between clinical malaria episodes; (iii) absence of other *Plasmodium* infections; and (iv) if female, the absence of pregnancy. The malaria diagnosis was conducted at the Hospital Universitário Júlio Muller (UFMT), Cuiabá, MT, from 2004 to 2013. This hospital is located in a region that is currently in the pre-elimination phase of malaria, and local *P*. *vivax* infection is considered highly improbable. All *P*. *vivax*-patients had traveled to an endemic area of malaria, where they were infected. After returning home, the *P*. *vivax*-patients were not re-exposed to *Plasmodium* infection, which was confirmed in the anamnesis by the clinician who treated the patients at the malaria reference center in Cuiabá. Those individuals were treated with CQ (25 mg/kg for 3 days) and PQ (0.5 mg/kg for 7 days) according to the guidelines of the Brazilian Ministry of Health.

### Blood collection and DNA extraction

*P*. *vivax* infection was confirmed by microscopic examination of Giemsa-stained thick blood smears that were evaluated by well-trained microscopists, according to the malaria diagnosis guidelines of the Brazilian Ministry of Health. Venous blood samples (5 mL, EDTA tubes) were collected at the time of each *P*. *vivax* episode. DNA was purified from the blood samples using the genomic DNA purification kit (Gentra Puregene, Minneapolis, MN, USA) according to the manufacturer’s protocols.

### Genotyping of CYP2D6 and CYP2C8 polymorphisms using Real-Time PCR

We genotyped five single-nucleotide polymorphisms (SNPs) in the *CYP2D6* gene (G1846A [rs3892097], G2988A [rs28371725], G3183A [rs59421388], C100T [rs1065852] and C1023T [rs28371706]) and two SNPs in the *CYP2C8* gene (G416A [rs11572080] and A805T [rs11572103]). To genotype the *CYP2D6*/*CYP2C8* genes by Real-Time PCR, we used specific hydrolysis probes for each SNP assay (Applied Biosystems, Foster City, CA, USA). All amplification reactions were performed in a total volume of 5 μL and in the presence of 2.5 μL Taqman*®* Universal PCR Master Mix 2x (Applied Biosystems, AB), 0.25 μL Genotyping Assay (AB), 1.25 μL water and 1 μL DNA (≈10 ng/μL). The cycling parameters for the PCR were as follows: initial denaturation at 95°C for 10 min, 50 cycles of 15 seconds at 92°C and 90 seconds at 60°C. Amplification and fluorescence detection were carried out using the ViiA 7 Real-Time PCR System (AB).

### CYP2D6 copy number assay

We determined the copy number of the *CYP2D6* gene by Real-Time PCR to evaluate the *CYP2D6* gene deletion and/or duplication using the Hs00010001_cn assay (AB). All amplification reactions were performed in the presence of 5.0 μL Taqman*®* Universal PCR Master Mix 2x (AB), 0.5 μL Copy Number Assay (AB), 0.5 μL Copy Number Reference Assay Human RNase P (AB), 3 μL water and 1 μL DNA (≈10 ng/μL). The cycling parameters used were as follows: 95°C for 10 minutes, followed by 40 cycles of 95°C for 15 seconds and 60°C for 60 seconds. Amplification was determined using the ViiA 7 Real-Time PCR System (AB).

### Microsatellites and MSP-1 genotyping

Eight loci of microsatellites (MS01, MS02, MS04, MS05, MS06, MS07, MS08, and MS11) and two loci of MSP1 (blocks 2 and 10) were amplified using specific primers and conditions as previously described [12]. For electropherogram analysis, the minimum peak height was set to 150 arbitrary fluorescence units (rFU). Additionally, we used the cut-off values for the minor peak detection of one-third the height of the predominant peak to exclude artifact peaks.

### Computational analysis of the effect of CYP2D6 polymorphisms on PQ metabolism

The structural effects of the CYP2D6 polymorphisms were assessed using mCSM-Stability [30] and DUET [31] as a way to shed light into the molecular mechanism of the mutation’s impact giving rise to a phenotype, as previously described [32–35]. These approaches are novel machine-learning algorithms that use the 3D structure to predict quantitatively the effects of point mutations [36]. The available crystal structure of human CYP2D6 was used in this analysis (PDB code 3TBG [37]). PQ was docked into the active site of the structure using AutoDock. The effect of the mutations was assessed in the context of the molecular interactions of the wild-type residue [38, 39], and mCSM and DUET were used to predict the effects of the mutations on protein stability. The potential effects of the mutations on flexibility were also assessed using the coarse-grained normal mode analysis server ENCoM [40].

### Statistical analysis

Fisher’s exact test or χ2 test was performed to compare the CYP2D6/CYP2C8 allele and the genotype frequencies or parasite genotype among groups of individuals differing in the number of relapses. Unadjusted odds ratios (ORs) were calculated with 95% confidence intervals (CIs) to determine the association between the genotypes and the risk of relapse. The statistical associations between the two groups defined according to the number of relapses and CYP2D6 mutant status were inferred using fitting logistic regression models. We also used logistic regression analysis to test the association between time to the first episode of recurrence of vivax malaria and CYP2C8 mutant status. Welch´s t-test and the Mann-Whitney U test were performed to compare the differences in average age, number of previous malaria episodes, parasitemia and time to relapse between single- and multiple-relapse groups. Statistical analysis was performed using R software (version 3.1.1). The Hardy-Weinberg equilibrium was calculated using the SNPassoc package from R software. *P*-value <0.05 was considered significant in all analyses. A correction for multiple testing was performed by multiplying the *P*-values by the number of the tests (Bonferroni correction).

## Results

### CYP2D6 Genotypes and Relapse in vivax malaria

To investigate whether variants of the cytochrome P-450 2D6 enzyme are associated with an increased risk of *P*. *vivax* relapse, five polymorphisms known to be responsible for low or null metabolic activity of CYP2D6 were analyzed in 46 patients who had relapsed *P*. *vivax* infections. According to the number of relapses observed during a follow-up period of 6 months, two groups were defined: the single-relapse group (characterized by a single episode of *P*. *vivax* relapse) and the multiple-relapse group (characterized by two to three episodes of relapses); both groups did not differ significantly in age, number of previous malaria episodes, parasitemia levels or time to the first episode of relapse (Table 1).

**Table 1:** Demographic and epidemiological data of individuals who were enrolled in this study.

Characteristics	Single-relapse(n = 28)	Multiple-relapse (n = 18)	*P*-value
Age, *years* (mean ± s.d.)	33.7 ± 14.7	35.4 ± 14.6	0.713^a^
Previous malaria episode, *n* (median ± s.d.)	2.0 ± 6.9	3.0 ± 3.7	0.445^b^
Parasitemia, *parasites/μL* (median ± s.d.)	1908.0 ± 4797.0	4085.0 ± 4834.0	0.377 ^b^
Time to the first relapse, *months* (median ± s.d.)	1.88 ± 1.13	1.71 ± 1.04	0.295 ^b^

Abbreviations: s.d., standard deviation; n, absolute number.

^a^Welch´s t-test.

^b^Mann-Whitney test.

**Fig 1:**
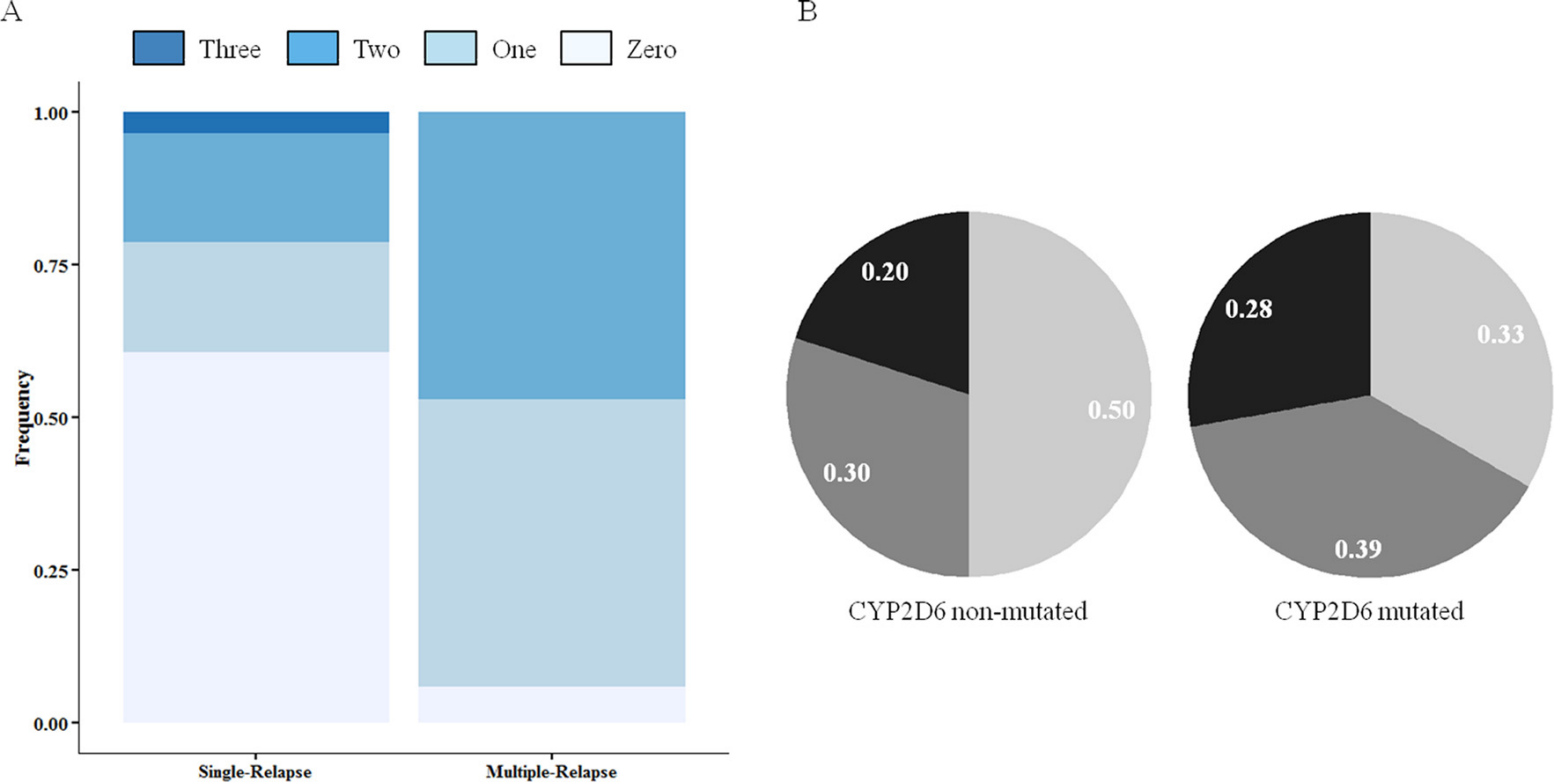
Frequency of CYP2D6 polymorphisms and parasite haplotype among *P*. *vivax-*infected patients who had single (n = 28) or multiple (n = 18) episodes of relapse. (A) The number of CYP2D6 polymorphisms is represented by the different intensity of color as specified in the legend. A simple logistic regression model shows a significant relationship between the mutant status for CYP2D6 and the increased number of relapses (OR, 12.4; 95% CI, 2.80–88.57; *P* = .003). (B) Frequency of parasite haplotype in patients without or with CYP2D6 mutation. Parasites were classified according to the number of markers containing identical alleles: *identical* in black (parasites showing all 10 identical markers); *related* in gray (8 to 9 identical markers); and *heterologous* in light gray (less than 8 identical markers).

Of the five SNPs genotyped in the *CYP2D6* gene, the most prevalent polymorphisms were C100T and G1846A, which are known to code for a significantly impaired enzyme (Table 2). Of note, for the C100T polymorphism, a significantly higher frequency of heterozygous and homozygous mutant genotypes was observed in individuals who experienced multiple-relapse infections (12/18 [66.7%] vs 7/28 [25.0%]; *P* = .007; Bonferroni-adjusted *P*_*c*_ = .049). Considering the presence of the mutated allele in any of the five nucleotide positions, a higher frequency of individuals with one or more polymorphic sites was observed in the multiple-relapse group compared with the single-relapse group (16/17 [94.1%] vs 11/28 [39.3%]; *P* = .0003; Bonferroni-adjusted *P*_*c*_ = .0021) (Fig 1A).

**Table 2:** Genotypes and allele frequencies of the *CYP2D6* gene in *P*. *vivax*-infected patients who had single or multiple episodes of relapse.

	Genotypes and Allele Frequencies n (%)				
**C100T (IM/PM)**^a^	***CC***	***CT***	***TT***	***CT*+*TT*** ^c^	***T*** ^e^
Single-relapse (n = 28)	21 (0.750)	2 (0.071)	5 (0.179)	7 (0.250)	0.214
Multiple-relapse (n = 18)	6 (0.333)	7 (0.389)	5 (0.278)	12 (0.667)	0.472
*P*			0.010^b^	0.007^d^	0.012^f^
OR (95% CI)				5.74 (1.39–27.06)	3.28 (1.31–8.18)
**C1023T (IM)**	***CC***	***CT***	***TT***	***CT*+*TT***	***T***
Single-relapse (n = 28)	25 (0.893)	2 (0.071)	1 (0.036)	3 (0.107)	0.071
Multiple-relapse (n = 18)	16 (0.889)	2 (0.111)	0 (0.000)	2 (0.111)	0.056
*P*			1.000	1.000	1.000
OR (95% CI)				1.04 (0.08–10.17)	0.77 (0.13–4.41)
**G1846A (PM)**	***GG***	***GA***	***AA***	***GA*+*AA***	***A***
Single-relapse (n = 28)	21 (0.750)	4 (0.143)	3 (0.107)	7 (0.250)	0.179
Multiple-relapse (n = 17)	8 (0.471)	7 (0.412)	2 (0.118)	9 (0.529)	0.324
*P*			0.098	0.107	0.187
OR (95% CI)				3.28 (0.79–14.68)	2.20 (0.81–5.93)
**G2988A (IM)**	***GG***	***GA***	***AA***	***GA*+*AA***	***A***
Single-relapse (n = 28)	28 (1.000)	0 (0.000)	0 (0.000)	0 (0.000)	0.000
Multiple-relapse (n = 18)	17 (0.944)	1 (0.056)	0 (0.000)	1 (0.056)	0.028
*P*			0.391	0.391	0.391
OR (95% CI)				NT	NT
**G3183A (IM)**	***GG***	***GA***	***AA***	***GA*+*AA***	***A***
Single-relapse (n = 28)	27 (0.964)	0 (0.000)	1 (0.036)	1 (0.036)	0.036
Multiple-relapse (n = 17)	17 (1.000)	0 (0.000)	0 (0.000)	0 (0.000)	0.000
*P*			1.000	1.000	0.525
OR (95% CI)				NT	NT

Abbreviations: CI, confidence interval; OR, odds ratio; NT, not testable.

^a^Predicted phenotype based on The Human Cytochrome P-450 (CYP) Allele Nomenclature Database (http://www.cypalleles.ki.se/): IM–intermediate metabolizer; PM–poor metabolizer.

^b^Comparison of the genotype frequencies between single-relapse and multiple-relapse groups; 2-tailed Fisher’s exact test.

^c^Frequency of the heterozygous and homozygous mutant genotypes.

^d^Comparison of carrier frequencies of the mutant allele; 2-tailed Fisher’s exact test.

^e^Frequency of the mutant allele.

^f^Comparison of the allele frequencies between single-relapse and multiple-relapse groups; 2-tailed Fisher’s exact test.

Because the number of *CYP2D6* gene copies may vary and alter the physiological levels of activity, we estimated the copy number of this gene in the study patients. All individuals had a single copy of *CYP2D6* (data not shown).

### CYP2D6 Variability and Parasite Genotype

Next, parasites from 24 patients (with samples available from their initial episode and relapses) were genotyped for the 10 molecular markers, and their haplotypes were correlated with the enzyme variants. We classified the parasites present in the different infections of the same patient as *identical* (when all of the markers have the same allele), *related* (8 to 9 markers with identical alleles), and *heterologous* (less than 8 markers with the same allele) [12]. As shown in Fig 1B, a high number of identical or related parasites was observed in individuals carrying the CYP2D6 mutated alleles (12/18 [67%] vs 5/10 [50%]). This difference was not statistically significant (*P* = .444).

### Effect of CYP2D6 Polymorphisms on the Metabolism of Primaquine

We carried out an in silico structural analysis of the two CYP2D6 polymorphisms (C100T and C1023T) that had a high prevalence among our samples to determine their effects specifically on PQ metabolism. The substitutions G1846A and G2988A occur in an intronic region and are associated with a splicing defect. Whereas G1846A occurs at the consensus sequence of the splice site of the 3rd intron of the *CYP2D6* gene, leading to a defective enzyme [41], the G2988A substitution is responsible for lower expression of the enzyme by quantitatively modulating the splicing events around exon/intron 6 [42].

**Fig 2:**
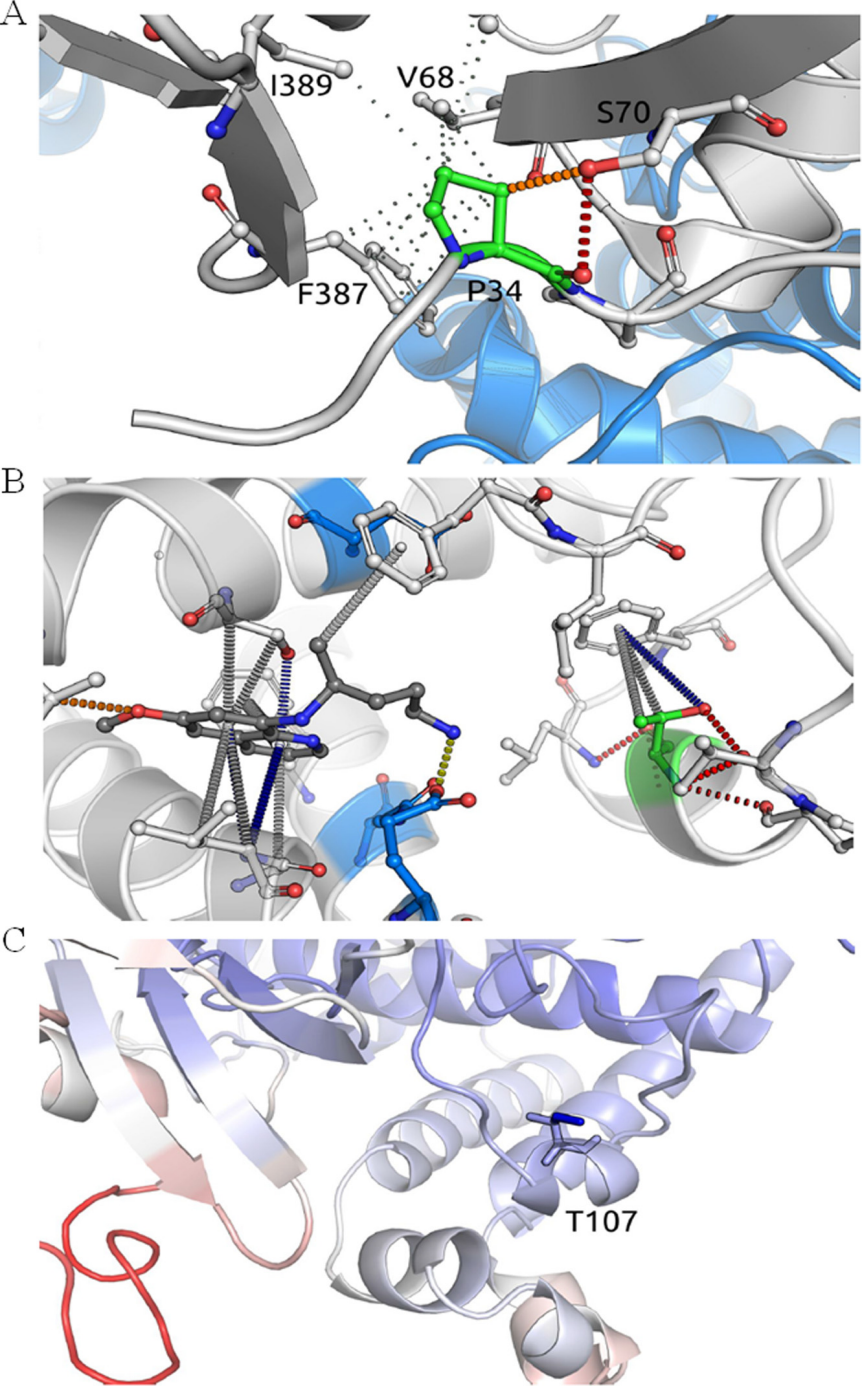
Analysis of the molecular interactions of polymorphic residues of CYP2D6 and their effects on the metabolism of primaquine. (A) The residue P34 has a buried side chain that is inserted into a predominantly hydrophobic environment (the hydrophobic interactions are depicted as gray dots) and is performing a main-chain to side-chain polar interaction with a neighboring beta strand (red dashes). The mutation P34S is predicted to destabilize the protein because it disrupts the local hydrophobic interaction network and affects the backbone rigidity. (B) The interactions for docked PQ (dark gray) and residue T107 (green). Threonine 107 is located in the vicinity of the PQ binding pocket (6.1 Å from PQ) and also nearby important catalytic residues (depicted in blue). (C) The mutation T107I results in the formation of increased local interactions, reducing CYP2D6 flexibility. Residues are colored based on their predicted effect on flexibility, ranging from more flexible (red) to less flexible (blue).

The mutation P34S (nucleotide substitution C100T) was predicted to be highly destabilizing by three different methods (ΔΔG mCSM-Stability: -1.984 Kcal/mol; SDM: -1.920 Kcal/mol; and DUET: -2.084 Kcal/mol). The residue P34 imparts rigidity to the backbone and is part of a hydrophobic interaction network, which is lost in the P34S mutation, leading to a greater degree of backbone freedom and destabilization of the enzyme (Fig 2A).

The mutation T107I (nucleotide substitution C1023T) was predicted to lead to an increase in protein stability (ΔΔG mCSM-stability: 0.258 Kcal/mol; SDM: 2.190 Kcal/mol; and DUET: 0.495 Kcal/mol). The residue T107 is localized in the vicinity of the PQ binding pocket, and the mutation T107I results in the formation of increased local hydrophobic interactions (Fig 2B). As a consequence, there is an increase in the protein stability that significantly reduces CYP2D6 flexibility, leading to reduced PQ metabolism (Fig 2C). This was supported by analysis using the ENCoM server, which predicted that the T107I mutation would reduce flexibility in this region.

### CYP2C8 Variability and Relapses by *Plasmodium vivax*

**Fig 3:**
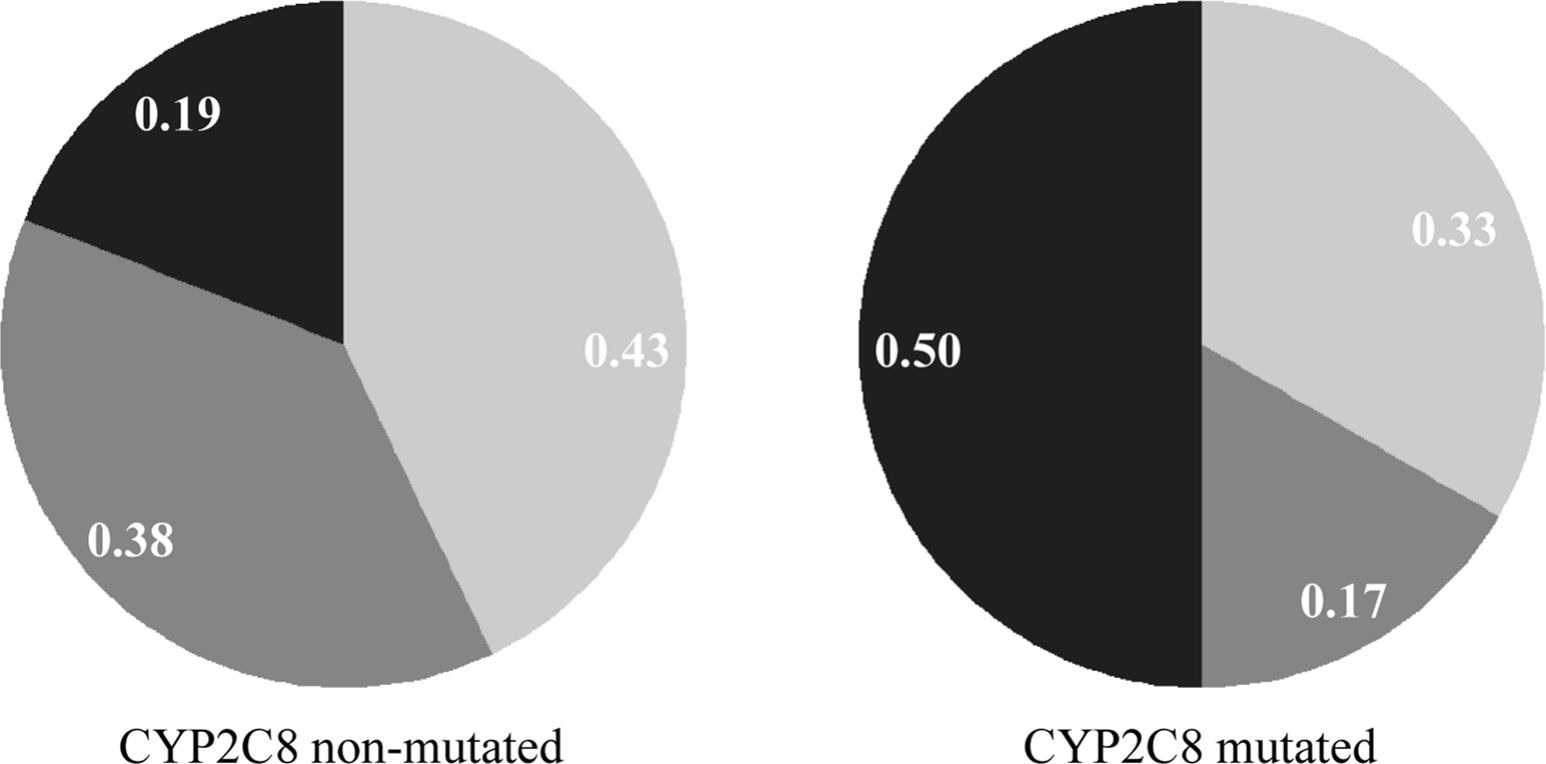
Analysis of the parasite haplotype and time to relapse in days for carriers of CYP2C8 polymorphisms. Frequency of parasite haplotype between patients without or with CYP2C8 mutation. Parasites were classified according to the number of identical markers: *identical* in black (10 identical markers); *related* in gray (8 to 9 identical markers); and *heterologous* in light gray (less than 8 identical markers).

To verify if polymorphisms that affect CYP2C8 functionality could also contribute to the observed *P*. *vivax* relapses, the individuals were genotyped for two polymorphisms that are associated with lower CQ metabolism. The CYP2C8*3 (G416A) allele was more frequent among the studied individuals, and there was no difference between the single- or multiple-relapse groups (6/27 [22.2%] and 6/18 [33.3%], respectively) of individuals carrying the mutated allele (*P* = 0.499) (Table 3). Next, we sought to investigate the relationship between the parasite genotype and CYP2C8 variability. A high proportion of identical parasites was found among mutated-enzyme carriers (3/6 [50%]). However, compared with the wild-type CYP2C8 carriers, the difference was not statistically significant (4/21 [19%]; *P* = .369) (Fig 3). Due to a limited sample size, this analysis could not be performed separately for single- and multiple-relapse groups. Additionally, the first episode of relapse was shorter in the group of patients who had a mutation in CYP2C8 (41.00 ± 30.80 days in the CYP2C8-mutated group vs 58.50 ± 37.37 days in the non-mutated group; *P* = .025). A higher proportion of mutated CYP2C8 subjects had *P*. *vivax* relapse, on average, within 42 days after the initial episode (8/15 [53.3%] under 42 days vs 6/31 [19.3%] above 42 days, *P* = .038) (S1 Fig). Hence, the odds of an early relapse were increased 4.8 times in patients whose mutation resulted in defective CYP2C8 metabolism (OR, 4.76; *P* = .023).

**Table 3:** CYP2C8 genotypes and allele frequencies in *P*. *vivax*-infected patients with different numbers of relapse.

	Genotype and Allele Frequencies n (%)^b^				
CYP2C8*2 (A805T)^a^	***AA***	***AT***	***TT***	***AT*+*TT***^d^	***T***^f^
Single-relapse (n = 28)	27 (0.964)	1 (0.036)	0 (0.000)	1 (0.036)	0.018
Multiple-relapse (n = 18)	16 (0.889)	2 (0.111)	0 (0.000)	2 (0.111)	0.056
*P*			0.552^c^	0.552^e^	0.559^g^
OR (95% CI)				3.37 (0.28–40.26)	3.23 (0.28–37.06)
CYP2C8*3 (G416A)	***GG***	***GA***	***AA***	***GA*+*AA***	***A***
Single-relapse (n = 27)	21 (0.778)	5 (0.185)	1 (0.037)	6 (0.222)	0.130
Multiple-relapse (n = 18)	12 (0.667)	5 (0.278)	1 (0.056)	6 (0.333)	0.194
*P*			0.745	0.499	0.554
OR (95% CI)				1.73 (0.37–8.17)	1.62 (0.52–5.09)

Abbreviations: CI, confidence interval; OR, odds ratio; NT, not testable.

^a^CYP2C8 allele nomenclature and the nucleotide change.

^b^The loci are in Hardy-Weinberg equilibrium in both groups (*P* > 0.05).

^c^Comparison of the genotype frequencies between single-relapse and multiple-relapse groups; 2-tailed Fisher’s exact test.

^d^Frequency of the heterozygous and homozygous mutant genotypes.

^e^Comparison of the allele frequencies between single-relapse and multiple-relapse groups; 2-tailed Fisher’s exact test.

^f^Frequency of the mutant allele.

^g^Comparison of carriers’ frequencies of the mutant allele; 2-tailed Fisher’s exact test.

## Discussion

Many factors may contribute to the success of drug therapies, including adherence to the prescribed therapy, correct or optimal dosing, general health status of the patient, interactions with other drugs, and the contribution of the parasite genetics particularly related to drug resistance. Another fundamental aspect that influences the treatment response is the way an individual metabolizes the drug. Previous studies, which had very few patients, suggested that polymorphisms in CYP2D6 might hinder malaria treatment and contribute to the relapse of *P*. *vivax* infections [16, 17, 43]. Aiming to confirm this hypothesis, we retrospectively analyzed the CYP2D6 genotype of patients who were not re-infected but experienced recurrent parasitemia. The strong evidence in favor of CYP2D6 enzyme variations on the outcome of malaria treatment comes from the association between the repeated relapses and the frequency of alleles associated with low PQ metabolism. Hence, the prevalence of CYP2D6 polymorphisms analyzed was approximately two times higher among subjects who had multiple episodes of relapse when compared with the single-relapse group. The current findings were corroborated by in silico analysis that showed that the structure of CYP2D6 could be disrupted by the polymorphisms studied in two different ways: destabilizing the enzyme structure and reducing the protein flexibility, especially around the PQ recognition site and catalytic residues.

Reinforcing the association between the PQ failure and the status of CYP2D6 activity, we found that a high amount of relapses in the CYP2D6 mutated-allele carriers was caused by parasites identical or related (defined here as homologous). These results seem to be consistent with the reactivation of homologous hypnozoites due to inefficacy of the PQ treatment. Accordingly, in a clinical trial of anti-relapse drugs, it was proposed that the homologous recurrence rate was the best predictor for comparing the efficacy of anti-hypnozoite drugs [44]. In fact, we and others have demonstrated that the majority of relapse episodes are caused by a parasite population distinct from the initial infection [12, 13, 45]. Nevertheless, the concept of the genetic profile of relapsing parasites is very complex and involves the following: (1) the occurrence of multiplicity of the infection, enabling the presence of rare alleles not detected either in the initial infection or in the relapses [12]; (2) the fluctuation of the frequency of circulating parasite clones at the initial infection and relapses [12, 46, 47]; and (3) previous infections as a source of heterologous hypnozoites [15]. While we cannot rule-out all of these possibilities in the present study, the last issue is unlikely because both groups (single- vs multiple-relapse groups) did not differ in the number of previous malaria episodes, reducing the possibility of bias in genetic characterization of the parasites.

Although the present study supports the role of the host CYP2D6 metabolizer status on vivax malaria treatment’s outcome, this study has some limitations. Firstly, the concentration of the parent drug and its main metabolite carboxyprimaquine (CPQ) in the plasma was not determined. Despite that, it is well established that the PQ therapeutic effects depend on the CYP2D6-generated metabolites [20, 23]. Secondly, because the treatment was not closely supervised, PQ treatment failure could be due to suboptimal dosing of the drug. Although the PQ dose was weight-based adjusted in a seven-day regimen, we cannot disregard the possibility of some patients' non-compliance to the treatment. While this could be responsible for a few cases of PQ treatment failure, the non-compliance could not explain the difference observed here between individuals who had a single or repeated episode of relapse. Additionally, adherence to the malaria treatment is usually high in different Brazilian localities (ranging from 67% to 86%) [48, 49]. Thirdly, CYP2D6 variations were not exhaustively explored in this study because the *CYP2D6* gene is highly polymorphic and is represented by more than 100 different alleles [50]. Owing to this complexity, the inference of a patient's CYP2D6 metabolic capacity or phenotype is a challenging task. Herein, we focused on five SNPs in the *CYP2D6* gene that frequently occur in the Brazilian population and are associated with a decreased drug metabolism phenotype [24]. Therefore, a more comprehensive analysis of the CYP2D6 genetic variation may add valuable information regarding the malaria treatment outcome.

For over half a century, PQ combined with CQ has been the standard radical curative regimen for vivax malaria. In contrast to PQ, CQ acts mainly on the blood stages of *P*. *vivax*, and CYP2C8 is the major enzyme involved in its metabolism. We evaluated the polymorphisms in CYP2C8 to consider the possibility that some recurrences may be due to CQ therapy failure associated with variation in CYP2C8. The results showed that 30% of patients have mutations in the CYP2C8 enzyme. As expected, the occurrence of polymorphisms in CYP2C8 conferred a lower CQ metabolism and did not differ between the single- and multiple-relapse groups because CQ does not eliminate the hypnozoite in the liver. Interestingly, patients who carried the CYP2C8 mutated alleles frequently had their first episode of recurrence within 42 days after the initiation of therapy. These early recurrences were associated with a high proportion of homologous parasites in the mutated-CYP2C8 carriers. Altogether, these findings support that the early recurrences in mutated-CYP2C8 patients could be due to blood-stage drug treatment failure associated with the impaired metabolism of CQ. Unfortunately, the plasma of individuals was not available, and thus, the plasma concentrations of CQ and its main metabolite (N-desethylchloroquine) could not be determined, which could give additional insights about CYP2C8 functionality. Beyond drug failure in mutated-carriers, another possibility is that the early recurrence could be the result of *P*. *vivax* resistance to CQ [1, 51]. However, CQ-resistance might not explain the proportion of identical parasites in the mutated-CYP2C8 carriers. Futures studies to elucidate the molecular background of parasites that are sensitive or resistant to CQ will help to clarify whether the early recurrence is due to CQ resistance or therapeutic failure.

In general, the main finding of this study indicates that polymorphisms in CYP2D6 are implicated in PQ treatment failure and may explain part of the *P*. *vivax* relapses. Additionally, the functional impairment of CYP2C8 may also contribute to therapeutic failure causing the recurrence of vivax infections. These results highlight the importance of pharmacogenetics to monitor the efficacy of antimalarial therapy and to design strategies for malaria elimination/eradication. In this context, the knowledge of individual genetic variation in enzymes involved in the metabolism of the antimalarial drugs might shed light on the type of *P*. *vivax* recurrence, i.e., recrudescence, relapse or new infection.

## Supporting Information

S1 Fig Description of the CYP genotypes, number of relapses and time to episode of relapse for 46 *P*. *vivax*-infected patients included in the present study. The individuals who were mutated in CYP2D6/CYP2C8 are indicated in black. For two individuals, some genotypes could not be determined (in gray). The number of relapses is indicated by the following colors: one (light green), two (green) and three (dark green). Patients who relapsed early (< 42 days after the initiation of therapy) are highlighted in light blue, and those who relapsed later (> 42 days) are indicated in dark blue. A simple logistic regression model shows a significant relationship between the mutant status for CYP2C8 and the time to the first episode of recurrence (OR, 4.76; 95% CI, 1.27–19.46; *P* = .023).
(TIF)
