# Correction: Variation in Human Cytochrome P-450 Drug-Metabolism Genes: A Gateway to the Understanding of *Plasmodium vivax* Relapses

**DOI:** 10.1371/journal.pone.0192534

**Published:** 2018-02-01

**Authors:** Ana Carolina Rios Silvino, Gabriel Luiz Costa, Flávia Carolina Faustino de Araújo, David Benjamin Ascher, Douglas Eduardo Valente Pires, Cor Jesus Fernandes Fontes, Luzia Helena Carvalho, Cristiana Ferreira Alves de Brito, Tais Nobrega Sousa

There is an error in Table 1. Please see the corrected Table 1 here.

**Table 1:** Demographic and epidemiological data of individuals who had been enrolled in the study.

Characteristics	Single-relapse (n = 28)	Multiple-relapse (n = 18)	*P* value
Age, *years* (mean ± s.d.)	33.7 ± 14.7	35.4 ± 14.6	0.713^a^
Previous malaria episode, *n* (median, IQR)	2 (1–6)	3 (2–6)	0.445^b^
Parasitemia, *parasites/μL* (median, IQR)	1908 (823–5100)	4085 (752–6909)	0.377 ^b^
Time to the first relapse, *months* (median, IQR)	1.88 (1.40–2.74)	1.71 (1.13–2.10)	0.295 ^b^

Abbreviations: s.d., standard deviation; IQR, interquartile range; n, absolute number

^a^Welch′s t test

^b^Mann-Whitney test
