# Supplementary figures and images for: Variation in Human Cytochrome P-450 Drug-Metabolism Genes: A Gateway to the Understanding of Plasmodium vivax Relapses
Source: PLoS One. 2016 Jul 28;11(7):e0160172. doi: 10.1371/journal.pone.0160172 (PMC4965052; doi:10.1371/journal.pone.0160172)

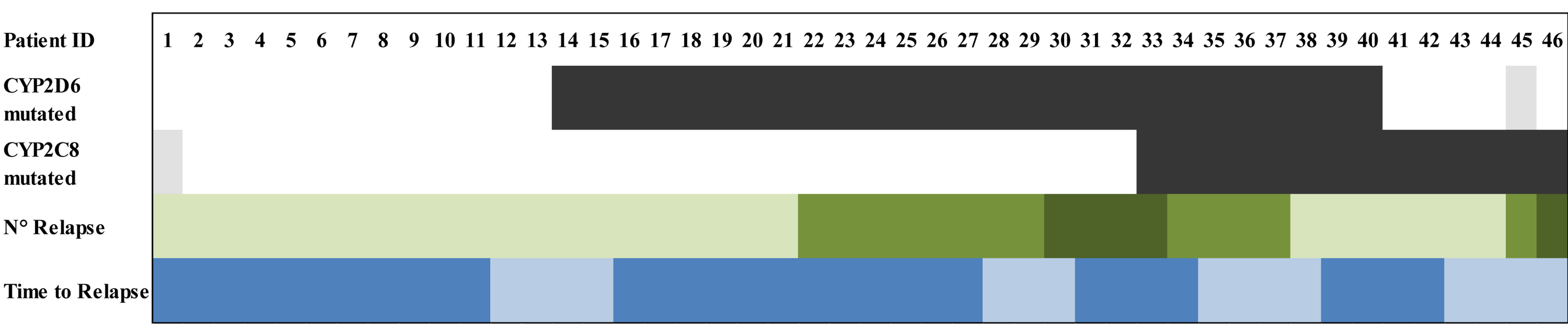

Supplement: S1 Fig — The individuals who were mutated in CYP2D6/CYP2C8 are indicated in black. For two individuals, some genotypes could not be determined (in gray). The number of relapses is indicated by the following colors: one (light green), two (green) and three (dark green). Patients who relapsed early (< 42 days after the initiation of therapy) are highlighted in light blue, and those who relapsed later (> 42 days) are indicated in dark blue. A simple logistic regression model shows a significant relationship between the mutant status for CYP2C8 and the time to the first episode of recurrence (OR, 4.76; 95% CI, 1.27–19.46; P = .023). (TIF) [file pone.0160172.s001.tif]
